# Association of prior depressive symptoms and suicide attempts with subsequent victimization: analysis of population-based data from the Adult Psychiatric Morbidity Survey

**DOI:** 10.1192/j.eurpsy.2020.50

**Published:** 2020-05-20

**Authors:** Vishal Bhavsar, Stephani L Hatch, Kimberlie Dean, Sally McManus

**Affiliations:** 1 Department of Health Services and Population Research, Section of Women’s Mental Health, Institute of Psychiatry, Psychology, and Neuroscience, King’s College London, London, United Kingdom; 2 South London and Maudsley NHS Foundation Trust, Maudsley Hospital, London, United Kingdom; 3 Department of Psychological Medicine, Institute of Psychiatry, Psychology, and Neuroscience, King’s College London, London, United Kingdom; 4 School of Psychiatry, University of New South Wales, Sydney, New South Wales, Australia; 5 Justice Health & Forensic Mental Health Network, Matraville, New South Wales, Australia; 6 National Center for Social Research, NatCen, London, United Kingdom

**Keywords:** Depression, epidemiology, intimate partner violence, sexual violence, victimization, workplace violence

## Abstract

**Background:**

Symptoms of mental disorder, particularly schizophrenia, predispose to victimization. Much less is known about the relationship between depressive symptoms and later victimization in the general population, the influence of these symptoms on types of subsequent victimization, or the role of symptom severity. We investigated this in nationally representative data from the United Kingdom.

**Methods:**

Data were from the Adult Psychiatric Morbidity Survey 2007. Multivariable logistic regressions estimated association between (a) prior depressive symptoms, (b) prior depressive symptoms with suicide attempt, and types of more recent victimization. Gender-specific associations were estimated using multiplicative interactions.

**Results:**

Prior depressive symptoms were associated with greater odds of any recent intimate partner violence (IPV), emotional IPV, sexual victimization, workplace victimization, any victimization, and cumulative victimization (adjusted odds ratio [aOR] for increasing types of recent victimization: 1.47, 95% confidence interval [CI]: 1.14, 1.89). Prior depressive symptoms with suicide attempt were associated with any recent IPV, emotional IPV, any victimization, and cumulative victimization (aOR for increasing types of recent victimization: 2.33, 95% CI: 1.22, 4.44). Self reported recalled data on previous depressive symptoms may have limited accuracy. Small numbers of outcomes for some comparisons results in imprecision of these estimates.

**Conclusions:**

Aside from severe mental illness such as schizophrenia, previous depressive symptoms in the general population are associated with greater subsequent victimization. Men and women with prior depressive symptoms may be vulnerable to a range of types of victimization, and may benefit from interventions to reduce this vulnerability.

## Introduction

Violence is a global public health challenge [1]. While symptoms of mental disorders have long been understood to be a potential consequence of violent victimization, recent research indicates such symptoms might also increase vulnerability to later victimization [2, 3]. However, much previous research has focused on mental disorders in help-seeking populations (e.g., Christ et al. [4], reviewed in Khalifeh et al. [5[), rather than symptoms of common mental disorders, such as depressed mood and suicidality, occurring in people who may not be seeking help. Victimization can occur in a variety of settings, such as in the context of an intimate relationship, or at the workplace. Victimization can involve emotional or sexual victimization, as well as physical harm. However, there has been limited assessment of potential associations of depressive symptoms with vulnerability to different types of victimization, or victimization occurring in different settings. Previous studies of intimate partner violence (IPV) in people with depressive symptoms have focused only on physical IPV [6–8], without examining emotional IPV, which involves recurrent criticism, verbal aggression and threats, and coercive or controlling behavior occurring within an intimate relationship. There has also been limited focus on types of victimization other than IPV, such as sexual victimization, defined by the World Health Organization as any sexual act against a person using coercion [9], and workplace victimization [10], defined by the European Commission as incidents where persons are abused, threatened, or assaulted in circumstances related to their work [10]. There is evidence that some individuals experience a disproportionately greater occurrence of victimization [11], and that different types of victimization are correlated [12]. However, few studies have examined if depressive symptoms increase risk of experiencing a range of victimization types, if there are differences between types of victimization in this association, or if depressive symptoms increase vulnerability over a continuum of cumulative victimization (i.e., whether association is similar when comparing those with no victimization versus one type of victimization, and those with one type of recent victimization versus two types of victimization, etc.). Experience of victimization varies between men and women, with women experiencing a greater burden of IPV, but studies suggesting greater physical victimization (specifically) among men [13]. This indicates that there could be different but overlapping risk factors for victimization experienced by men, compared to women. Depressive symptoms may also predispose to some types of victimization more than others. Feelings of fear, helplessness, and entrapment in IPV relationships may predispose both to depressive symptoms and to further IPV victimization [14]. In contrast, depressive symptoms may increase the likelihood of work absence, due to the influence of depressive symptoms on motivation and the execution of job roles, thus resulting in lower risk of workplace victimization [15]. It is possible therefore that any greater likelihood of workplace victimization experienced by people with previous depressive symptoms is less than that for IPV, because of the association of depressive symptoms with greater work absence. Epidemiological studies on victimization in mental illness have examined birth cohorts (therefore, only including individuals of a specific age) [16, 17], household surveys of urban settings [18], and clinical samples [19], but have rarely evaluated nationally representative data on depressive symptoms [20].

Therefore, there is a need for national population-based studies on what factors influence vulnerability to a range of types of victimization in people with depressive symptoms. Few studies on increased IPV risk in depression have accounted for the shared correlation of both depression [21, 22] and IPV in adulthood with childhood abuse [23, 24]. It is also not known whether any association between prior depressive symptoms and subsequent victimization is confounded by prior nonviolent adverse life events, such as homelessness, running away from home, or by violent behavior. Finally, there has been limited assessment of possible bias introduced by differences in recall of prior traumatic events between those with and without depression at the time of research interview.

In this study, we tested the relationship between prior depressive symptoms (occurring more than 1 year ago) and recent victimization in nationally representative data from the United Kingdom. We hypothesized

1. association between prior depressive symptoms and recent victimization;
2. that greater severity of prior depressive symptoms, indicated by the report of prior suicide attempt, would be accompanied by greater risk of recent victimization; and
3. stronger association of prior depressive symptoms with recent IPV compared to recent workplace victimization.

## Methods

### Sample details

We analyzed data from the Adult Psychiatric Morbidity Survey 2007 (APMS), which draws on a representative sample of household residents in the United Kingdom [25]. The survey was commissioned by NHS Digital and carried out by the National Center for Social Research (NatCen) and University of Leicester. A multistage stratified probability sampling design was adopted. The sampling frame was the Post Office’s small user Postcode Address File, covering private households in the United Kingdom. The first stage of sampling involved the selection of primary sampling units (PSUs) and the second stage involved selecting addresses within PSUs. People living in communal establishments were not surveyed. When interviewers made contact at an address, one resident aged 16 or over was randomly selected for interview. The questionnaire was administered using a combination of face-to-face and self-completion computer-assisted interviewing, covering physical health, mental health, service use, religion, social capital, discrimination, violence, and abuse. Fieldwork took place between October 2006 and December 2007 with 7,403 adults.

### Ethical standards

Ethical approval was obtained for APMS 2007 from Research Ethics Committees of the National Research Ethics Service appropriate for nonclinical populations. The authors assert that all procedures contributing to this work comply with the ethical standards of the relevant national and institutional committees on human experimentation and with the Helsinki Declaration of 1975, as revised in 2008.

### Measures

#### Prior depressive symptoms and prior suicide attempt

Information on previous episodes of depression was collected in the Common Mental Disorders section of the APMS questionnaire, and information on suicide attempts was taken from the Suicidal Thoughts section. To ascertain prior depressive symptoms, we used information from an item assessing any previous episodes of feeling sad, miserable, or depressed, and another item enquiring for the age the first of these episodes occurred. We used this information and respondent age to derive a dichotomous variable to indicate any prior depressive symptoms occurring a year or more ago. Information on prior suicide attempt was gathered by asking participants if they had made an attempt to take their own life prior to the last year. These variables were combined to create a three-level variable for reporting (a) neither prior depressive symptoms nor prior suicide attempt, (b) prior depressive symptoms alone, and (c) prior depressive symptoms with prior suicide attempt.

#### Recent victimization events

Self-completion items in the Domestic Violence and Abuse section of the APMS questionnaire assessed recent IPV, in the form of experiencing, in the previous 12 months, a partner or ex-partner:

- pushing, holding, or pinning you down, or slapping you; choking or trying to strangle you; using a weapon against you; or using some other kind of force against you (for recent physical IPV) or
- threatening you with a weapon; threatening to kill you; or issuing threats causing fear (for recent emotional IPV).

These were used to derive variables for any recent IPV, recent emotional IPV, and recent physical IPV. Recent sexual victimization was assessed with self-report items enquiring whether respondents had, in the previous 12 months, experienced any nonconsensual sexual touching or sexual intercourse, in the Stressful Life Events section of the APMS questionnaire. Information on recent workplace victimization was measured using face-to-face interview data on recent experience of violence at work, with a reference period of 6 months, in the Stressful Life Events section of the APMS questionnaire. Victimization variables analyzed in this study were not mutually exclusive. Based on these variables, we derived a binary indicator for recent victimization of any type, and an ordered categorical variable for number of different types of recent victimization experienced. This score theoretically ranged from 0 to 4, however, in the observed data ranged from 0 to 3.

#### Lifetime nonviolent adverse life events

Bereavement, separation, serious interpersonal difficulties, being sacked or made redundant, joblessness/job-searching for longer than 1 month, or major financial crisis were assessed by checklist. Based on this variable, we created a binary variable reflecting any nonviolent adverse life events in the respondents’ lifetime [26]. These items were contained in the Stressful Life Events section of the APMS questionnaire.

#### Childhood physical or sexual abuse

Physical victimization during childhood was assessed by asking whether the participant had, before the age of 16, experienced severe physical beating by a stepparent, parent, or carer. Sexual abuse was evaluated by asking respondents if they had experienced someone talking in a sexual way to them without consent before the age of 16, if they had experienced nonconsensual sexual touching before the age of 16, or if they were subject to nonconsensual sexual intercourse before the age of 16. These items were used to derive a binary variable reflecting childhood abuse.

#### Covariates

Age was measured in years and grouped into age groups of 16–24, 25–44, and 45 and above for description, and included in regression models as a continuous variable. Gender was dichotomized, and self-ascribed ethnicity classified into U.K. census categories, and then further categorized into Black, Asian, White British, White non-British, and mixed/other categories for this analysis. Social class was classified according to the Office for National Statistics’ National Statistics Socio-Economic Classification [27], dropping the military occupational category because of small numbers. Employment status at the time of interview was grouped into unemployed or not unemployed. Marital status at interview was categorized into single, married/cohabiting, divorced/separated, and widowed. Highest educational qualification was classified into no qualifications, General Certificate of Secondary Education (GCSE) (reflecting schooling until around 16 years of age), A levels (schooling until 18 years of age), and attaining a degree. A binary item measuring lifetime perpetration of violence was based on asking participants whether they had ever assaulted or deliberately hit someone in the context of physical fight [28]. Drug use was measured by an item for use of an illicit drug in the lifetime (illicit drugs included cannabis, amphetamines, cocaine, crack, ecstasy, heroin, acid, magic mushrooms, tranquilizers, amyl nitrite, anabolic steroids, and glue), and hazardous use of alcohol in the previous year was measured using the Alcohol Use Disorders Identification Test (AUDIT) scale [29], with a cutoff of 8. Neighborhood deprivation was measured by linking the respondent’s postcode at interview to a publicly available census-derived deprivation index, the Index of Multiple Deprivations 2007. To limit identifiability of individual respondents, this information was made available as a five-level variable, for the quintile of deprivation for each respondent, based on their address. Information on current symptoms of depression was collected using the Revised Clinical Interview Schedule [30]. Current depression was identified using diagnostic criteria from the 10th International Classification of Diseases [31].

### Analysis

We examined distribution of prior depressive symptoms, and prior depressive symptoms with suicide attempt (both reported to have occurred at least 1 year prior to interview), and any recent IPV (in the last 12 months), recent emotional IPV (in the last 12 months), recent physical IPV (in the last 12 months), recent sexual victimization (6 months), recent workplace victimization (6 months), any recent victimization, and experiencing two or more types of recent victimization, by all analyzed covariates, with counts and survey-weighted proportions.

Based on the epidemiological literature, we conceptualized prior depressive symptoms, prior suicide attempt, and later victimization as potentially influenced by the following potential confounders: age, gender, educational attainment, childhood abuse, use of drugs and alcohol, lifetime nonviolent adverse life event, and perpetration of violence, presenting this as a directed acyclic graph (see the Supplementary Material) [32]. Other possible socioeconomic confounders from the graph (marital status, social class, ethnic group, income, and neighborhood deprivation) were evaluated for inclusion based on the amount of deviation from the unadjusted estimate for association between prior depressive symptoms and recent victimization, using a difference in the adjusted association of 10% or greater compared to the crude estimate [33] to indicate evidence of possible confounding (see Table S4). On this basis, educational attainment, childhood abuse, lifetime nonviolent adverse life event, violence perpetration, lifetime drug use, and hazardous alcohol use were included in fully adjusted models, together with age and gender.

Crude associations between each included covariate and each victimization type were estimated using survey-weighted logistic regressions. For multivariable modeling, survey-weighted logistic regression analyses in Stata 14 [34] were used to estimate associations between prior depressive symptoms alone, and prior depressive symptoms with suicide attempt, and any recent IPV, emotional IPV, physical IPV, sexual victimization, workplace victimization, and any recent victimization of any type. Ordinal logistic regression models were used to estimate association between prior depressive symptoms alone, and prior depressive symptoms with suicide attempt, and a greater number of types of recent victimization experienced. All models were estimated overall, and for men and women using multiplicative interaction terms for gender, to derive male- and female-specific estimates. In order to test for a trend in associations of victimization types with prior depressive symptoms alone, and prior depressive symptoms with suicide attempt, likelihood ratio tests were used to test if a linear term provided better fit than an indicator variable. We report these *p* values for strength of evidence against the appropriateness of including a linear term, based on the overall sample, for each victimization type, in Table 2. Final model estimates for covariates are reported in Table S1.

Finally, we carried out sensitivity analyses. We examined the impact of missing data on our results by comparing prevalence of victimization outcomes in those included in the analysis with those excluded due to missing data, stratified into those without previous depressive symptoms, those with prior depressive symptoms without suicide attempt, and those with previous depressive symptoms and suicide attempt. We also compared final model estimates with estimates from 15 imputed datasets, generated using multiple imputation by chained equations, combining estimates from imputed datasets using Rubin’s rules [35]. Our primary analysis was a complete case analysis. Model estimates based on complete cases assume data are missing completely at random. Briefly, multiple imputation allows examination of the impact of missing data on model results, under the assumption that missing data are related to variables that are observed in the dataset (data missing at random), but cannot account for data which are missing due to factors that are not observed in the data (data missing not at random) [36]. We also estimated models restricted to data from those without current depression, in order to examine a possible role for different recall accuracy for victimization between those with and without depression at the time of interview, and the influence of prior victimization on our results, by estimating models restricted to those without a history of childhood abuse.

## Results

### Sample characteristics

Table 1 describes counts and survey-weighted percentages on the study sample. The total sample consisted of 7,403 respondents, of whom 48.6% (*n =* 3,197) were male, 50% (4,387) were above 45, and 25.6% (2,278) reported attaining no qualifications. The prevalence of childhood abuse was 15.8% (1,200). Around a quarter of the sample (24.1%, 1,603) reported hazardous use of alcohol, and a quarter (25.7%, 1,637) reported lifetime drug use. Nine-tenths of the sample (92.2%, 6,946) reported at least one lifetime nonviolent adverse life event. Lifetime perpetration of violence was reported by 18.2% of the sample (1,268). Diagnostic criteria for current depression were met by 3% of the total sample (255). Data were complete on the analyzed variables in 7,068 (95%) participants.

**Table 1.** Description (in the form of counts and survey-weighted percentages) of prior depressive symptoms alone, and prior depressive symptoms with prior suicide attempt, by each victimization type and covariate in the survey sample (*n* = 7,403).

	Neither previous depressivesympto ms nor suicide attempt		Prior depressive symptoms		Prior depressive symptoms with prior suicide attempt		Row total	
	Count (%)^a^		Count (%)^a^		Count (%)^a^		Count (%)^a^	
Any recent IPV								
No	4,517	(95.5)	2,355	(93.2)	157	(83.8)	7,029	(94.4)
Yes	207	(4.5)	143	(6.8)	24	(16.2)	374	(5.6)
Recent emotional IPV								
No	4,563	(96.7)	2,379	(94.3)	162	(86.6)	7,104	(95.7)
Yes	161	(3.3)	119	(5.7)	19	(13.4)	299	(4.3)
Recent physical IPV								
No	4,606	(97.3)	2,429	(96.7)	167	(90.7)	7,363	(97.0)
Yes	118	(2.7)	69	(3.3)	14	(9.3)	40	(3.0)
Recent sexual victimization								
No	4,711	(99.7)	2,476	(98.6)	176	(96.3)	7,376	(99.3)
Yes	13	(0.3)	22	(1.4)	5	(3.7)	27	(0.7)
Recent workplace victimization								
No	4,716	(99.8)	2,480	(99.1)	180	(99.3)	7,376	(99.5)
Yes	8	(0.2)	18	(0.9)	1	(0.7)	27	(0.5)
Any recent victimization								
No	4,499	(95.0)	2,329	(91.8)	153	(80.9)	6,981	(93.6)
Yes	225	(5.0)	169	(8.2)	28	(19.1)	422	(6.4)
Greater than two types of recent victimization								
No	4,650	(98.5)	2,447	(97.5)	172	(93.6)	7,269	(98.0)
Yes	74	(1.5)	51	(2.5)	9	(6.4)	134	(2.0)
Age (years)								
16–24	374	(14.8)	175	(12.7)	19	(16.6)	568	(14.2)
25–44	1,463	(33.6)	907	(39.6)	78	(43.3)	2,448	(35.9)
45+	2,887	(51.6)	1,416	(47.7)	84	(40.1)	4,387	(50.0)
Gender								
Male	2,137	(50.1)	1,000	(46.4)	60	(37.9)	3,197	(48.9)
Female	2,587	(49.9)	1,498	(53.6)	121	(62.1)	4,206	(51.4)
Educational qualifications								
No qualifications	1,618	(28.9)	613	(19.7)	47	(21.7)	2,278	(25.6)
GCSE	1,311	(30.0)	727	(30.2)	65	(39.4)	2,103	(30.3)
A level	575	(14.5)	341	(15.3)	22	(13.8)	938	(14.8)
Degree	1,104	(24.3)	771	(33.2)	41	(22.6)	1,916	(27.2)
Missing	116	(2.3)	46	(1.8)	6	(2.6)	168	(2.1)
Childhood abuse								
No	4,097	(87.3)	2,008	(80.2)	98	(53.8)	6,203	(84.2)
Yes	627	(12.7)	490	(19.8)	83	(46.2)	1,200	(15.8)
Hazardous use of alcohol								
No	3,738	(76.9)	1,923	(74.0)	128	(67.1)	5,789	(75.7)
Yes	976	(22.9)	574	(25.9)	53	(32.9)	1,603	(24.1)
Missing	10	(0.2)	1	(0.0)	0	(0.0)	11	(0.1)
Lifetime drug use								
Yes	885	(21.6)	665	(31.2)	87	(52.8)	1,637	(25.6)
No	3,802	(77.7)	1,826	(68.6)	92	(46.2)	5,720	(73.9)
Missing	37	(0.7)	7	(0.2)	2	(1.0)	46	(0.6)
Lifetime nonviolent life events								
No	354	(9.4)	98	(4.9)	5	(2.9)	457	(7.8)
Yes	4,370	(90.6)	2,400	(95.1)	176	(97.1)	6,946	(92.2)
Lifetime perpetration of violence								
No	3,994	(83.5)	1,981	(78.1)	109	(57.6)	6,084	(81.1)
Yes	690	(15.6)	508	(21.6)	70	(41.4)	1,268	(18.2)
Missing	40	(0.8)	9	(0.3)	2	(1.0)	51	(0.7)
Current depressive episode								
No	4,547	(96.9)	2,441	(97.8)	160	(90.3)	7,148	(97.0)
Yes	177	(3.1)	57	(2.2)	21	(9.7)	255	(3.0)
Column total	4,724	(64.2)^b^	2,498	(33.5)^b^	181	(23.2)^b^	7,403	(100)

Abbreviations: IPV, intimate partner violence.

a Column percentages.

b Row percentages.

#### Prior depressive symptoms and prior suicide attempt

The overall prevalence of prior depressive symptoms (i.e., reported to have occurred at least 12 months ago) was 33.5% (2,498) and prior depressive symptoms with suicide attempt was 2.3% (181). Respondents reporting prior depressive symptoms and prior depressive symptoms with suicide attempt were more likely to be female. Childhood abuse was more prevalent in those with previous depressive symptoms (19.8%, 490) and prior depressive symptoms with suicide attempt (46.2%, 83) than those with neither prior depressive symptoms nor suicide attempt (12.7%, 627). Hazardous use of alcohol was more common among those with prior depressive symptoms (25.9%, 574) and prior depressive symptoms with suicide attempt (53, 32.9%), than those with neither prior depressive symptoms nor suicide attempt (976, 22.9%). Lifetime drug use was more commonly reported in those with prior depressive symptoms (665, 31.2%) and prior depressive symptoms with suicide attempt (87, 52.8%), than those with neither prior depressive symptoms nor suicide attempt (885, 21.6%). Lifetime nonviolent adverse life events were more common in those with prior depressive symptoms (2,400, 95.1%) and prior depressive symptoms with prior suicide attempt (176, 97.1%), than those with neither prior depressive symptoms nor suicide attempt (4,370, 90.6%). Lifetime perpetration of violence was more likely to be reported by people with prior depressive symptoms (508, 21.6%), and those with prior depressive symptoms with suicide attempt (70, 41.4%), compared to those without (690, 15.6%).

#### Recent victimization

The prevalence of any recent IPV, recent emotional IPV, recent physical IPV, recent sexual victimization, and recent workplace victimization, and any recent victimization were all greater among those with prior depressive symptoms, and among those with prior depressive symptoms with suicide attempt, than those with neither. For example, 19.1% (28) respondents with prior depressive symptoms and suicide attempt reported any recent victimization, compared to 8.2% (169) of those with prior depressive symptoms only and 5% (225) of those with neither depressive symptoms nor suicide attempt.

### Multivariable estimates for association of prior depressive symptoms, and prior suicide attempt, with types of recent victimization

In relation to our first hypothesis, prior depressive symptoms were statistically associated with all recent victimization types in the overall sample, except recent physical IPV, before adjustments (see Table 2). After adjustment for potential confounders, prior depressive symptoms alone remained associated with recent IPV (odds ratio [OR]: 1.31, 95% confidence intervals [CI]: 1.01, 1.69), recent emotional IPV (OR: 1.48, 95% CI: 1.12, 1.97), recent sexual victimization (OR: 2.90, 95% CI: 1.37, 6.11), recent workplace victimization (OR: 3.33, 95% CI: 1.37, 8.12), any recent victimization (OR: 1.43, 95% CI: 1.12, 1.83), and cumulative victimization (OR for a greater number of types of recent victimization: 1.47, 95% CI: 1.14, 1.89). After adjustment, prior depressive symptoms with suicide attempts remained associated with any recent IPV (OR: 2.19, 95% CI: 1.19, 4.00), recent emotional IPV (2.44, 95% CI: 1.26, 4.75), recent sexual victimization (OR: 5.85, 95% CI: 1.51, 22.63), any recent victimization (OR: 2.48, 95% CI: 1.38, 4.45), and cumulative victimization (OR: 2.33, 95% CI: 1.22, 4.44). Tests for trend in associations of prior depressive symptoms alone and prior depressive symptoms with suicide attempt suggested a linear trend in the strength of associations for all outcomes (see Table 2), in support of our second hypothesis.

**Table 2.** Association (odds ratios, with 95% confidence intervals) between prior depressive symptoms alone, prior depressive symptoms with prior suicide attempt (both occurring more than 1 year ago), each type of recent victimization, based on the overall analytic sample, and for men and women.

	Unadjusted		Fully adjusted	
	Prior depressive symptoms	Prior depressive symptoms and suicide attempt	Prior depressive symptoms	Prior depressive symptoms and suicide attempt
Recent IPV				
Overall	1.57 (1.24, 1.98)	4.41 (2.66, 7.33)	1.31 (1.01, 1.69)	2.19 (1.19, 4.00)
Men	1.92 (1.34, 2.77)	3.62 (1.44, 9.12)	1.61 (1.09, 2.39)	1.73 (0.58, 5.20)
Women	1.32 (0.95, 1.82)	4.60 (2.52, 8.41)	1.10 (0.78, 1.55)	2.39 (1.22, 4.68)
Recent emotional IPV				
Overall	1.75 (1.34, 2.27)	4.81 (2.72, 8.50)	1.48 (1.12, 1.97)	2.44 (1.26, 4.75)
Men	2.46 (1.61, 3.76)	4.38 (1.58, 12.13)	2.11 (1.35, 3.29)	2.16 (0.67, 7.04)
Women	1.30 (0.90, 1.87)	4.69 (2.36, 9.33)	1.10 (0.75, 1.62)	2.48 (1.17, 5.25)
Recent physical IPV				
Overall	1.19 (0.86, 1.66)	3.92 (1.98, 7.76)	0.91 (0.64, 1.31)	1.58 (0.75, 3.32)
Men	1.27 (0.76, 2.13)	0.87 (0.12, 6.55)	0.98 (0.56, 1.70)	0.33 (0.04, 2.85)
Women	1.12 (0.73, 1.72)	5.36 (2.58, 11.10)	0.87 (0.59, 1.36)	2.36 (1.07, 5.22)
Recent sexual victimization				
Overall	3.31 (1.52, 7.19)	11.99 (3.68, 39.08)	2.90 (1.37, 6.11)	5.85 (1.51, 22.63)
Men	5.69 (1.62, 19.92)	18.34 (2.62, 128.44)	4.93 (1.52, 15.95)	9.12 (1.06, 78.36)
Women	2.01 (0.68, 5.95)	8.79 (1.99, 38.89)	1.77 (0.61, 5.13)	4.09 (0.90, 18.69)
Recent workplace victimization				
Overall	4.13 (1.64, 10.41)	3.24 (0.39, 27.05)	3.33 (1.37, 8.12)	2.20 (0.27, 17.87)
Men	4.17 (1.37, 12.72)	–	3.23 (1.10, 9.48)	–
Women	4.55 (0.95, 21.90)	–	3.59 (0.75, 17.19)	–
Any recent victimization				
Overall	1.71 (1.37, 2.14)	4.84 (2.99, 7.96)	1.43 (1.12, 1.83)	2.48 (1.38, 4.45)
Men	2.26 (1.60, 3.18)	5.21 (2.35, 11.53)	1.91 (1.32, 2.77)	2.62 (0.98, 7.01)
Women	1.33 (0.97, 1.82)	4.46 (2.44, 8.15)	1.10 (0.79, 1.54)	2.31 (1.17, 4.54)
Cumulative recent victimization				
Overall	1.71 (1.37, 2.13)	4.86 (2.97, 7.94)	1.47 (1.14, 1.89)	2.33 (1.22, 4.44)
Men	2.25 (1.60, 3.16)	4.85 (2.31, 10.16)	1.89 (1.31, 2.74)	2.41 (0.95, 6.12)
Women	1.33 (0.97, 1.82)	4.68 (2.45, 8.94)	1.12 (0.80, 1.56)	2.57 (1.23, 5.38)

The reference group for all estimates is reporting neither prior depressive symptoms nor prior suicide attempt. All estimates are based on 7,068 individuals with complete data on the final modeled variables.

Abbreviations: IPV, intimate partner violence.

Overall, models are adjusted for age, gender, educational attainment, childhood abuse, hazardous alcohol use, lifetime drug use, lifetime nonviolent adverse life events (in the form of either serious illness/assault to a relative, bereavement, separation, serious interpersonal difficulties, being sacked or made redundant, joblessness/job-searching for longer than 1 month, or major financial crisis), and lifetime perpetration of violence. Cumulative recent victimization estimates are from ordinal logistic regression models. Estimates for men and women are from models including a multiplicative interaction term for gender. Likelihood ratio tests indicated statistical evidence for a linear trend in ORs for prior depressive symptoms alone and prior depressive symptoms with suicide attempt. The *p* values for superior fit of nontrend model are as follows: any IPV: 0.2987, emotional IPV: 0.5776, physical IPV: 0.1156, workplace victimization: 0.1030, sexual victimization: 0.9208, any recent victimization: 0.3703, and cumulative victimization: 0.3703.

#### Associations in men and women

Confidence intervals for estimates in men and women overlapped, suggesting insufficient statistical evidence for differences in association between men and women. Adjusted associations of prior depressive symptoms alone with each victimization outcome were greater in magnitude among men, compared to women, with the exception of physical IPV, where the OR for women was 0.87 (95% CI: 0.59, 1.36) and 0.98 (95% CI: 0.56, 1.70) for men, and for workplace victimization, where the OR for women was 3.59 (95% CI: 0.75, 17.19) and 3.23 (95% CI: 1.10, 9.48) for men Table 2. Associations of prior depressive symptoms with suicide attempt with each type of recent victimization were stronger in women than men for recent IPV, recent emotional IPV, recent physical IPV, and cumulative victimization, but stronger in men than women for recent sexual victimization and any recent victimization. Estimates for workplace victimization were not produced due to low numbers.

### Sensitivity analyses

Estimates of association based on data restricted to those who did not report childhood abuse and among those who did not meet diagnostic criteria for depression at the time of interview were similar to our main results (Table 3). Chi-squared comparisons did not indicate significant differences in the prevalence of victimization types among excluded and included records, with the exception of recent sexual victimization which was more prevalent in excluded cases than those included (*p* < 0.001, Table S2). Estimates from multiple imputation did not differ in direction for any outcomes, but there was some attenuation of most fully adjusted estimates (Table S3).

**Table 3.** Association (odds ratios, with 95% confidence intervals) between prior depressive symptoms alone, and prior depressive symptoms with prior suicide attempt, with recent victimization types, restricted to those without depressive episode at the time of interview and those without a history of childhood abuse.

	Unadjusted		Fully adjusted	
	Prior depressive symptoms only	Prior depressive symptoms and prior suicide attempt	Prior depressive symptoms only	Prior depressive symptoms and prior suicide attempt
Recent IPV				
In those with no childhood abuse^a^	2.02 (1.51, 2.68)	5.03 (2.44, 10.37)	1.76 (1.30, 2.39)	2.90 (1.15, 7.30)
Without current depressive episode^b^	1.63 (1.28, 2.08)	4.37 (2.52, 7.59)	1.34 (1.03, 1.75)	1.99 (1.04, 3.82)
Recent emotional IPV				
In those with no childhood abuse^a^	2.39 (1.72, 3.34)	6.32 (2.87, 13.95)	2.14 (1.52, 3.02)	3.83 (1.47, 9.94)
Without current depressive episode^b^	1.83 (1.39, 2.42)	4.80 (2.58, 8.95)	1.54 (1.15, 2.08)	2.27 (1.11, 4.62)
Recent physical IPV				
In those with no childhood abuse^a^	1.41 (0.94, 2.11)	2.03 (0.65, 6.31)	1.13 (0.74, 1.74)	0.89 (0.25, 3.16)
Without current depressive episode^b^	1.27 (0.90, 1.78)	3.85 (1.81, 8.18)	0.95 (0.66, 1.37)	1.37 (0.61, 3.07)
Recent sexual victimization				
In those with no childhood trauma^a^	3.89 (1.21, 12.51)	21.20 (3.33, 135.06)	3.81 (1.15, 12.67)	20.68 (2.71, 157, 83)
Without current depressive episode^b^	3.82 (1.63, 8.95)	12.61 (3.21, 49.56)	3.32 (1.47, 7.51)	5.32 (1.09, 25.93)
Recent workplace victimization				
In those with no childhood abuse^a^	2.88 (1.01, 8.19)	–	2.59 (0.92, 7.35)	–
Without current depressive episode^b^	3.84 (1.51, 9.78)	3.49 (0.42, 29.18)	3.01 (1.22, 7.38)	2.25 (0.28, 18.31)
Any recent victimization				
In those with no childhood abuse^a^	2.08 (1.58, 2.75)	5.46 (2.73, 10.94)	1.84 (1.37, 2.47)	3.43 (1.41, 8.35)
Without current depressive episode^b^	1.76 (1.40, 2.22)	4.80 (2.84, 8.13)	1.46 (1.13, 1.88)	2.28 (1.21, 4.29)
Cumulative recent victimization				
In those with no childhood abuse^a^	2.08 (1.58, 2.74)	5.30 (2.71, 10.36)	1.85 (1.38, 2.48)	3.37 (1.40, 8.11)
Without current depressive episode^b^	1.76 (1.40, 2.22)	4.81 (2.81, 8.21)	1.47 (1.14, 1.89)	2.33 (1.22, 4.44)

The reference group for all estimates is reporting neither prior depressive symptoms nor prior suicide attempt.

Abbreviations: IPV, intimate partner violence.

a Based on 5,911 participants.

b Based on 6,829 participants.

## Discussion

### Summary of findings

Prior depressive symptoms were associated with any recent IPV, emotional IPV, sexual victimization (all in the previous 12 months), workplace victimization (in the previous 6 months), and cumulative recent victimization, supporting our first hypothesis. Associations of prior depressive symptoms with suicide attempt were greater in magnitude than prior depressive symptoms alone, in support of our second hypothesis. Associations of prior depressive symptoms with workplace victimization were greater in magnitude than for IPV, in disagreement with our third hypothesis. Although estimates for association between prior depressive symptoms alone with recent victimization were generally greater in magnitude in men than women (with the exception of recent physical IPV, where estimates for men and women were similar), the extent of this varied between types of victimization.

### Previous literature

Our study extends analyses of APMS data demonstrating cross-sectional association between IPV and psychiatric disorders [37], and that different types of victimization may be correlated over the lifecourse [38]. Our findings accord with some evidence that people with psychiatric disorders experience greater subsequent victimization. However, previous studies have focused on clinical populations with severe mental disorders [39, 40], not sampled the general population for controls [33, 41], and not accounted for perpetration [42–44]. Lehrer et al. [45] found association between depression and subsequent physical IPV in American adolescent girls in nationally representative data. However, as well as limited representativeness for the general population, they also did not account for drug use, perpetration, or socioeconomic information other than parental education. In prospective data from an HIV prevention trial in Eastern Cape Province, South Africa [46], depressive symptoms were associated with subsequent relationship abuse in women, but not men. There were a range of adjustments made in the study; however, the study was focused on HIV-affected individuals, and emotional abuse was not captured, which may explain weaker findings in men in this study. A study of nearly 500 pregnant women in Nicaragua [47] found crude association between depressive symptoms and continued abuse, but reported frequencies only, and did not adjust for confounders. A study in Uppsala, Sweden, compared depressed adolescent females with controls on psychosocial outcomes in adulthood, including physical and verbal IPV, adjusting for socioeconomic disadvantage, parental conflict, and disruptive behavior [7]. This study found IPV at follow-up was around 3.5 times commoner in those with depression at baseline; however, this did not account for alcohol or drug use, and representativeness was limited. In a study of rural schools in North Carolina, the United States, Fohshee et al. [6] found depressed girls were 1.4 times more likely to report subsequent sexual victimization, but did not find this relationship in boys. We are unaware of examinations of association between depressive symptoms and later sexual victimization in general population data, although studies have found higher occurrence of sexual victimization toward people with severe mental illness [48, 49].

Our finding that prior depressive symptoms predict workplace victimization is consistent with a small number of previous studies on workplace bullying [50]. Finne et al. [51] found Norwegian workers with anxiety were more likely to report workplace bullying at follow-up 5 years later; however, statistical evidence was found for men, not women, consistent with stronger associations found in our analysis in men compared to women. Kivimaki et al. [52] assessed the prospective relationship between workplace bullying and subsequent depression in a Finnish occupational cohort, but also found unadjusted “reverse” associations between depression at baseline and later depression, reporting that those with depression were around 2.5 times more likely to report workplace bullying at follow-up 2 years later. No studies have compared workplace victimization and IPV as outcomes in people with prior depressive symptoms, as far as we are aware.

### Strengths and limitations

We examined our hypotheses in a large, nationally representative, general population-based sample, allowing generalization of our findings to the English setting. Data were 95% complete, and sensitivity analyses suggested limited impact of missing data on our inferences. Association between prior depressive symptoms and recent victimization was evident even among those without childhood abuse, helping to limit the possibility of reverse causality affecting our results. Our hypotheses focused on self-reported depressive symptoms, rather than clinical depressive disorder, and our results should not be generalized to clinical depressive disorders. The sampling frame did not include institutional residents or homeless individuals, limiting generalizability. Assessment of prior depressive symptoms, by asking if respondents had experienced episodes of feeling sad, miserable, or depressed more than 1 year ago, was imprecise, and could have been more subject to differences in recall sensitivity between participants. No information was available on number, duration, and severity of prior depressive symptoms, although stronger associations for prior depressive symptoms with suicide attempt could indicate a dose-response relationship with severity of prior depressive symptoms. Although our data were collected at a single time point, variables investigated were separated in time. Nevertheless, information on prior depressive symptoms and IPV could have incorporated measurement error—accuracy of reporting IPV may have differed between those with and without prior depressive symptoms. There were small numbers of participants reporting recent sexual victimization and workplace victimization, leading to imprecise estimates, and these associations should assessed in samples with higher frequency of these outcomes. Self-report information on prior depressive symptoms may also have introduced error—individuals who had frequent experiences of IPV and other types of trauma could have been more sensitive to recalling or describing prior depressive symptoms, or suicide attempts. Risk factors for sexual or workplace victimization and IPV which were also causes of prior depressive symptoms could have been left out of models because they were not measured, or incompletely handled due to poor measurement. For example, we were not able to use information on prior experiences of IPV or sexual victimization in adulthood, although we were able to adjust for childhood abuse. Systematic differences in probability of overreporting IPV have been reported between men and women [53], although mechanisms underlying this, such as the reporting of IPV by men as a way to excuse their own violent behavior, remain speculative [54]. In particular, although it is theoretically possible that we overestimated the prevalence of IPV in men because of over-reporting of perpetration-type events, the survey data did not contain information on IPV perpetration, limiting our ability to test this.

### Explanations

Typically, the consistent overlap between mental disorders and victimization has been explained by a causal relationship between victimization and later mental disorder. However, a reverse relationship is also possible, and has been relatively underexplored in the literature. Depressive symptoms could increase vulnerability in social and workplace situations and influence a person’s ability or motivation to remove themselves from risky environments. Individuals with evident depressive symptoms, or suicide attempt, may be considered easy targets by potential perpetrators, due to their perceived vulnerability or lack of credibility in the event they report victimization—this has not been researched, as far as we are aware. Depressive symptoms are also associated with increased use of alcohol and drugs, and longitudinal studies are clearly required which measure intervening drug/alcohol use, in order to clarify the role of substances in this relationship. Given that IPV may increase risk of later depression [14], the impact of depressive symptoms on social relationship trajectories could contribute to enduring patterns of depressive symptoms and experience of IPV over the life course.

In our study, prior depressive symptoms remained associated with IPV even when physical IPV was removed, suggesting that these characteristics could increase risk of IPV through mechanisms involving emotional control, decision-making, and negotiation of relationships. On the other hand, the crude association between prior depressive symptoms and physical IPV was small, and attenuated nearly completely on adjustments—this is consistent with one previous prospective study of 79 young American couples suggested that depressive symptoms in women predicted psychological, but not physical partner aggression [55]. The reasons for this finding in our study are unclear—aside from a chance effect, it is possible that those who report physical IPV as well as emotional IPV were atypical of the broader population exposed to IPV, resulting in different patterns of associations with depressive symptoms. Depressive symptoms and suicide attempt may each act to increase emotional tension and strife in relationships, increasing emotional IPV, but might simultaneously act to reduce physical victimization by potential perpetrators, as the victim might be considered more vulnerable and unable to defend themselves, or because they spend less time in situations where they might experience victimization. Differing mechanisms linking depressive symptoms to emotional and physical IPV have not been explored as far as we know. Suicide attempt is common in people diagnosed with depression, personality disorders [56], but also in people in the general population who may not be in contact with mental health services [57]. In our study, the item capturing prior suicide attempt item may have been a reflection of impulsivity, depressive symptoms, or use of drugs or alcohol (although we adjusted for the latter in fully adjusted estimates). The possible impact of suicide attempt on risk of experiencing subsequent victimization deserves further study. Finally, our third hypothesis for weaker associations between depressive symptoms and workplace victimization was rejected, and further investigation of the impact of depressive symptoms on workplace victimization may also be warranted.

## Conclusions

Both men and women with prior depressive symptoms, with and without suicide attempt, may be vulnerable to a range of subsequent victimization types, and may benefit from interventions to reduce this vulnerability. Our findings suggest the specific importance of enquiring about new onset victimization in people with a history of depressive symptoms, or suicide attempt, rather than only focusing on early life trauma [58]. Prospective studies, evaluating type, setting, and perpetrators involved in victimization, are necessary for policy recommendations to be made.

## Data Availability

Data used in this study is available to download for research from the UK Data Service at https://beta.ukdataservice.ac.uk/datacatalogue/studies/study?id=6379.

## Supplementary material

For supplementary material accompanying this paper visit http://dx.doi.org/10.1192/j.eurpsy.2020.50. click here to view supplementary material
