# Supplementary material for: Association of prior depressive symptoms and suicide attempts with subsequent victimization: analysis of population-based data from the Adult Psychiatric Morbidity Survey
Source: Eur Psychiatry. 2020 May 20;63(1):e51. doi: 10.1192/j.eurpsy.2020.50 (PMC7355179; doi:10.1192/j.eurpsy.2020.50)
Supplement: Supplementary file 1 [file S0924933820000504sup.zip › S0924933820000504sup001.docx]

**SUPPLEMENTARY MATERIAL**

| Table S1. Fully adjusted model estimates for associations of prior depressive symptoms alone, and prior depressive symptoms with prior self-harm, and recent victimisation types, any recent victimisation, and cumulative victimisation. Models take account of survey design and are based on the final analytic sample for the study (n=7068). Age was included as a continuous linear variable in all models. Estimates for cumulative victimisation are from ordinal logistic regression. | | | | | | | | | | | | | | | | | | | | | |
| --- | --- | --- | --- | --- | --- | --- | --- | --- | --- | --- | --- | --- | --- | --- | --- | --- | --- | --- | --- | --- | --- |
|  | Any recent IPV | | | Recent emotional IPV | | | Recent physical IPV | | | Recent sexual victimisation | | | Recent workplace victimisation | | | Any recent victimisation | | | Cumulative victimisation | | |
|  | OR | CI | | OR | CI | | OR | CI | | OR | CI | | OR | CI | | OR | CI | | OR | CI | |
| Prior depressive symptoms and suicide attempt | | | | | | | | | | | | | | | | | | | | | |
| Neither prior depressive symptoms nor suicide attempt | 1.00 |  |  | 1.00 |  |  | 1.00 |  |  | 1.00 |  |  | 1.00 |  |  | 1.00 |  |  | 1.00 |  |  |
| Prior depressive symptoms only | 1.31 | 1.01 | 1.69 | 1.48 | 1.12 | 1.97 | 0.91 | 0.64 |  | 2.90 | 1.37 | 6.11 | 3.33 | 1.37 | 8.12 | 1.43 | 1.12 | 1.83 | 1.44 | 1.13 | 1.83 |
| Prior depressive symptoms with prior suicide attempt | 2.19 | 1.19 | 4.00 | 2.44 | 1.26 | 4.75 | 1.58 | 0.75 | 3.32 | 5.85 | 1.51 | 22.63 | 2.20 | 0.27 | 17.87 | 2.48 | 1.38 | 4.45 | 2.56 | 1.40 | 4.67 |
| Age | 0.97 | 0.96 | 0.97 | 0.97 | 0.96 | 0.98 | 0.96 | 0.95 | 0.97 | 0.94 | 0.91 | 0.97 | 0.98 | 0.95 | 1.00 | 0.96 | 0.96 | 0.97 | 0.96 | 0.96 | 0.97 |
| Gender |  |  |  |  |  |  |  |  |  |  |  |  |  |  |  |  |  |  |  |  |  |
| Male | 1.00 |  |  | 1.00 |  |  | 1.00 |  |  | 1.00 |  |  | 1.00 |  |  | 1.00 |  |  | 1.00 |  |  |
| Female | 1.31 | 1.01 | 1.70 | 1.24 | 0.94 | 1.63 | 1.63 | 1.13 | 2.35 | 0.64 | 0.31 | 1.30 | 0.33 | 0.14 | 0.80 | 1.11 | 0.87 | 1.42 | 1.13 | 0.88 | 1.44 |
| Educational attainment | | |  |  |  |  |  |  |  |  |  |  |  |  |  |  |  |  |  |  |  |
| No qualifications | 1.00 |  |  | 1.00 |  |  | 1.00 |  |  | 1.00 |  |  | 1.00 |  |  | 1.00 |  |  | 1.00 |  |  |
| GCSE | 1.03 | 0.71 | 1.48 | 0.92 | 0.61 | 1.39 | 1.00 | 0.62 | 1.61 | 1.82 | 0.64 | 5.18 | 4.67 | 0.55 | 39.41 | 1.11 | 0.78 | 1.57 | 1.10 | 0.77 | 1.57 |
| A level | 1.12 | 0.73 | 1.72 | 1.00 | 0.62 | 1.61 | 1.24 | 0.71 | 2.18 | 1.40 | 0.43 | 4.63 | 3.22 | 0.31 | 33.28 | 1.19 | 0.79 | 1.80 | 1.18 | 0.78 | 1.79 |
| Degree | 1.02 | 0.70 | 1.49 | 0.89 | 0.59 | 1.36 | 1.24 | 0.74 | 2.08 | 0.98 | 0.30 | 3.25 | 7.97 | 1.04 | 61.16 | 1.19 | 0.82 | 1.72 | 1.18 | 0.81 | 1.71 |
| Childhood abuse | | |  |  |  |  |  |  |  |  |  |  |  |  |  |  |  |  |  |  |  |
| No | 1.00 |  |  | 1.00 |  |  | 1.00 |  |  | 1.00 |  |  | 1.00 |  |  | 1.00 |  |  | 1.00 |  |  |
| Yes | 1.87 | 1.37 | 2.56 | 1.91 | 1.36 | 2.68 | 2.10 | 1.43 | 3.09 | 7.94 | 3.30 | 19.10 | 1.97 | 0.91 | 4.28 | 2.19 | 1.65 | 2.92 | 2.23 | 1.67 | 2.97 |
| Hazardous use of alcohol | | |  |  |  |  |  |  |  |  |  |  |  |  |  |  |  |  |  |  |  |
| No | 1.00 |  |  | 1.00 |  |  | 1.00 |  |  | 1.00 |  |  | 1.00 |  |  | 1.00 |  |  | 1.00 |  |  |
| Yes | 1.50 | 1.14 | 1.99 | 1.42 | 1.04 | 1.93 | 1.66 | 1.14 | 2.40 | 0.98 | 0.48 | 2.00 | 1.64 | 0.71 | 3.78 | 1.47 | 1.13 | 1.90 | 1.48 | 1.14 | 1.92 |
| Lifetime drug use | | |  |  |  |  |  |  |  |  |  |  |  |  |  |  |  |  |  |  |  |
| No | 1.00 |  |  | 1.00 |  |  | 1.00 |  |  | 1.00 |  |  | 1.00 |  |  | 1.00 |  |  | 1.00 |  |  |
| Yes | 1.12 | 0.83 | 1.53 | 1.06 | 0.76 | 1.49 | 1.19 | 0.81 | 1.74 | 0.59 | 0.27 | 1.29 | 0.74 | 0.31 | 1.77 | 1.02 | 0.76 | 1.37 | 1.03 | 0.77 | 1.38 |
| Lifetime non-violent adverse events | | | |  |  |  |  |  |  |  |  |  |  |  |  |  |  |  |  |  |  |
| No | 1.00 |  |  | 1.00 |  |  | 1.00 |  |  | 1.00 |  |  | 1.00 |  |  | 1.00 |  |  | 1.00 |  |  |
| Yes | 1.83 | 0.99 | 3.37 | 1.95 | 0.97 | 3.92 | 1.80 | 0.78 | 4.18 | 0.78 | 0.21 | 3.00 | 1.01 | 0.15 | 6.91 | 1.61 | 0.90 | 2.85 | 1.62 | 0.92 | 2.86 |
| Lifetime perpetration | |  |  |  |  |  |  |  |  |  |  |  |  |  |  |  |  |  |  |  |  |
| No | 1.00 |  |  | 1.00 |  |  | 1.00 |  |  | 1.00 |  |  | 1.00 |  |  | 1.00 |  |  | 1.00 |  |  |
| Yes | 3.04 | 2.29 | 4.02 | 2.71 | 2.00 | 3.68 | 4.69 | 3.18 | 6.92 | 1.21 | 0.50 | 2.91 | 2.38 | 1.03 | 5.49 | 2.90 | 2.23 | 3.76 | 2.95 | 2.28 | 3.84 |

| Table S2. Comparison of the categorical distribution of victimisation indicators in included records, with records excluded from complete case analysis due to missing data, stratified by prior depressive symptoms and suicide attempt. Brackets enclose column percentages, weighted for survey design. Also shown are chi-squared estimates and chi-squared p values. All chi-squared comparisons were on one degree of freedom. | | | | | | | | | |
| --- | --- | --- | --- | --- | --- | --- | --- | --- | --- |
|  | Neither previous depression suicide attempt | | | Previous depression only | | | Previous depression with suicide attempt | | |
|  | Excluded | Included | *X*^2^, p | Excluded | Included | *X*^2^, p | Excluded | Included | *X*^2^, p |
| Any previous IPV in the previous 12 months |  |  |  |  |  |  |  |  |  |
| No | 255(91.9) | 4262(95.7) |  | 52(84.3) | 2303(93.3) |  | 6(0.0) | 151(83.3) |  |
| Yes | 18(8.1) | 189(4.3) | 3.38, 0.066 | 4(15.7) | 139(6.7) | 0.21, 0.644 | 0(0.0) | 24(16.7) | 0.95, 0.330 |
| Emotional IPV in the previous 12 months |  |  |  |  |  |  |  |  |  |
| No | 260(94.9) | 4303(96.8) |  | 52(84.3) | 2327(94.5) |  | 6(0.0) | 156(86.3) |  |
| Yes | 13(5.1) | 148(3.2) | 1.61, 0.204 | 4(15.7) | 115(5.5) | 0.72, 0.398 | 0(0.0) | 19(13.7) | 0.7278, 0.394 |
| Physical IPV in the previous 12 months |  |  |  |  |  |  |  |  |  |
| No | 265(95.7) | 4341(97.4) |  | 54(89.5) | 2375(96.9) |  | 69(0.0) | 161(90.5) |  |
| Yes | 8(4.3) | 110(2.6) | 0.2226, 0.637 | 2(10.5) | 67(3.1) | 0.14, 0.709, | 0(0.0) | 14(9.5) | 0.52, 0.471 |
| Sexual violence |  |  |  |  |  |  |  |  |  |
| No | 273(100.0) | 4438(99.7) |  | 53(85.1) | 2423(98.9) |  | 6(0.0) | 170(96.2) |  |
| Yes | 0(0.0) | 13(0.3) | 0.80, 0.371 | 3(14.9) | 19(1.1) | 13.15, *<0.001* | 0(0.0) | 5(3.8) | 0.18, 0.675 |
| Workplace violence |  |  |  |  |  |  |  |  |  |
| No | 273(100.0) | 4443(99.8) |  | 56(0.0) | 2424(99.1) |  | 6(0.0) | 174(99.3) |  |
| Yes | 0(0.0) | 8(0.2) | 0.49, 0.483 | 0(0.0) | 18(0.9) | 0.42, 0.519 | 0(0.0) | 1(0.7) | 0.03, 0.853 |
| Any recent victimisation |  |  |  |  |  |  |  |  |  |
| No | 255(91.9) | 4244(95.2) |  | 51(79.9) | 2278(92.1) |  | 6(0.0) | 147(80.4) |  |
| Yes | 18(8.1) | 207(4.8) | 2.1403, 0.143 | 5(10.2) | 164(7.9) | 0.43, 0.514 | 0(0.0) | 28(19.6) | 1.14, 0.287 |

| Table S3. Association (odds ratios, with 95% confidence intervals) between prior depressive symptoms alone, and prior depressive symptoms with prior suicide attempt (both occurring more than one year ago) and each type of recent victimisation, based on the combination of 15 multiply imputed datasets, and for men and women. The reference group for all estimates is reporting neither prior depressive symptoms nor prior suicide attempt. | | | | |
| --- | --- | --- | --- | --- |
|  | Unadjusted | | Fully adjusted | |
| Recent IPV | Prior depressive symptoms | Prior depressive symptoms and suicide attempt | Prior depressive symptoms | Prior depressive symptoms and suicide attempt |
| Overall | 1.33(1.06, 1.65) | 3.34(2.12, 5.24) | 1.09(0.87, 1.38) | 1.66(1.02, 2.71) |
| Men | 1.42(1.06, 1.90) | 2.42(1.03, 5.70) | 1.16(0.85, 1.58) | 1.23(0.50, 3.03) |
| Women | 1.26(0.97, 1.64) | 3.81(2.27, 6.41) | 1.06(0.81, 1.40) | 1.93(1.10, 3.39) |
| Recent emotional IPV |  |  |  |  |
| Overall | 1.42(1.11, 1.81) | 3.32(2.01, 5.48) | 1.19(0.92, 1.53) | 1.68(0.98, 2.86) |
| Men | 1.55(1.13, 2.14) | 2.58(1.02, 6.52) | 1.32(0.95, 1.85) | 1.36(0.52, 3.59) |
| Women | 1.33(0.99, 1.78) | 3.71(2.08, 6.62) | 1.11(0.82, 1.51) | 1.87(1.01, 3.46) |
| Recent physical IPV |  |  |  |  |
| Overall | 1.11(0.82, 1.50) | 3.27(1.84, 5.82) | 0.86(0.63, 1.19) | 1.42(0.76, 2.75) |
| Men | 1.04(0.68, 1.60) | 0.66(0.09, 4.82) | 0.78(0.50, 1.23) | 0.28(0.04, 2.12) |
| Women | 1.15(0.81, 1.64) | 4.70(2.57, 8.59) | 0.95(0.65, 1.38) | 2.18(1.13, 4.21) |
| Recent sexual victimisation |  |  |  |  |
| Overall | 3.22(1.62, 6.40) | 10.30(3.63, 29.19) | 2.74(1.35, 5.57) | 5.03(1.65, 15.32) |
| Men | 4.40(2.00, 9.67) | 12.50(2.76, 56.63) | 4.42(1.96, 10.00) | 7.47(1.52, 36.72) |
| Women | 2.44(1.07, 5.57) | 9.21(2.59, 32.76) | 1.83(0.78, 4.29) | 3.78(0.99, 14.39) |
| Recent workplace victimisation |  |  |  |  |
| Overall | 4.28(1.86, 9.85) | 3.28(0.41, 26.32) | 3.34(1.43, 7.80) | 1.99(0.24, 16.75) |
| Men | 7.16(2.92, 17.56) | 9.99(1.12, 81.16) | 5.05(2.02, 12.62) | 5.49(0.64, 47.30) |
| Women | 2.37(0.82, 6.84) | 9.99(1.23, 81.16) | 1.85(0.63, 5.43) | 5.49(0.64, 47.30) |
| Any recent victimisation |  |  |  |  |
| Overall | 1.45(1.18, 1.78) | 3.66(2.39, 5.59) | 1.20(0.96, 1.49) | 1.85(1.17, 2.95) |
| Men | 1.74(1.33, 2.27) | 3.53(1.72, 7.26) | 1.45(1.10, 1.93) | 1.87(0.86, 4.05) |
| Women | 1.26(0.98, 1.63) | 3.72(2.24, 6.19) | 1.04(0.79, 1.35) | 1.85(1.06, 3.20) |
| Cumulative recent victimisation |  |  |  |  |
| Overall | 1.45(1.18, 1.78) | 3.66(2.39, 5.58) | 1.21(0.97 1.50) | 1.90(1.20, 3.01) |
| Men | 1.73(1.33, 2.25) | 3.33(1.63, 6.82) | 1.44(1.09, 1.91) | 1.83(0.80, 3.73) |
| Women | 1.27(0.98, 1.63) | 3.83(2.31, 6.37) | 1.06(0.81, 1.38) | 2.00(1.16, 3.45) |
| Overall models are adjusted for age, gender, educational attainment, childhood abuse, hazardous alcohol use, lifetime drug use, lifetime non-violent trauma (in the form of either serious illness/assault to a relative, bereavement, separation, serious interpersonal difficulties, being sacked or made redundant, joblessness/job-searching for longer than one month, or major financial crisis), and lifetime perpetration of violence. Cumulative recent victimisation estimates are from ordinal logistic regression models. Estimates for men and women are from models including a multiplicative interaction term for gender. | | | | |

| Table S4. Deviation of crude estimate for association between prior depressive symptoms and any recent victimisation, upon adjustment for each socioeconomic indicator. | | | | | |
| --- | --- | --- | --- | --- | --- |
|  | Coefficient for prior depressive symptoms | Coefficient for prior depressive symptoms and prior suicide attempt | Total | Change | Percentage change |
| Unadjusted | 0.53 | 1.51 | 2.04 |  |  |
| Adjusted for social class | 0.58 | 1.49 | 2.07 | 0.03 | 1.47 |
| Adjusted for ethnic group | 0.56 | 1.56 | 2.12 | 0.08 | 3.92 |
| Adjusted for income | 0.56 | 1.51 | 2.07 | 0.03 | 1.47 |
| Adjusted for neighbourhood deprivation | 0.55 | 1.49 | 2.04 | 0.00 | 0.00 |
| Adjusted for marital status | 0.53 | 1.42 | 1.95 | -0.09 | -4.41 |
