# Supplementary figures and images for: Association of prior depressive symptoms and suicide attempts with subsequent victimization: analysis of population-based data from the Adult Psychiatric Morbidity Survey
Source: Eur Psychiatry. 2020 May 20;63(1):e51. doi: 10.1192/j.eurpsy.2020.50 (PMC7355179; doi:10.1192/j.eurpsy.2020.50)

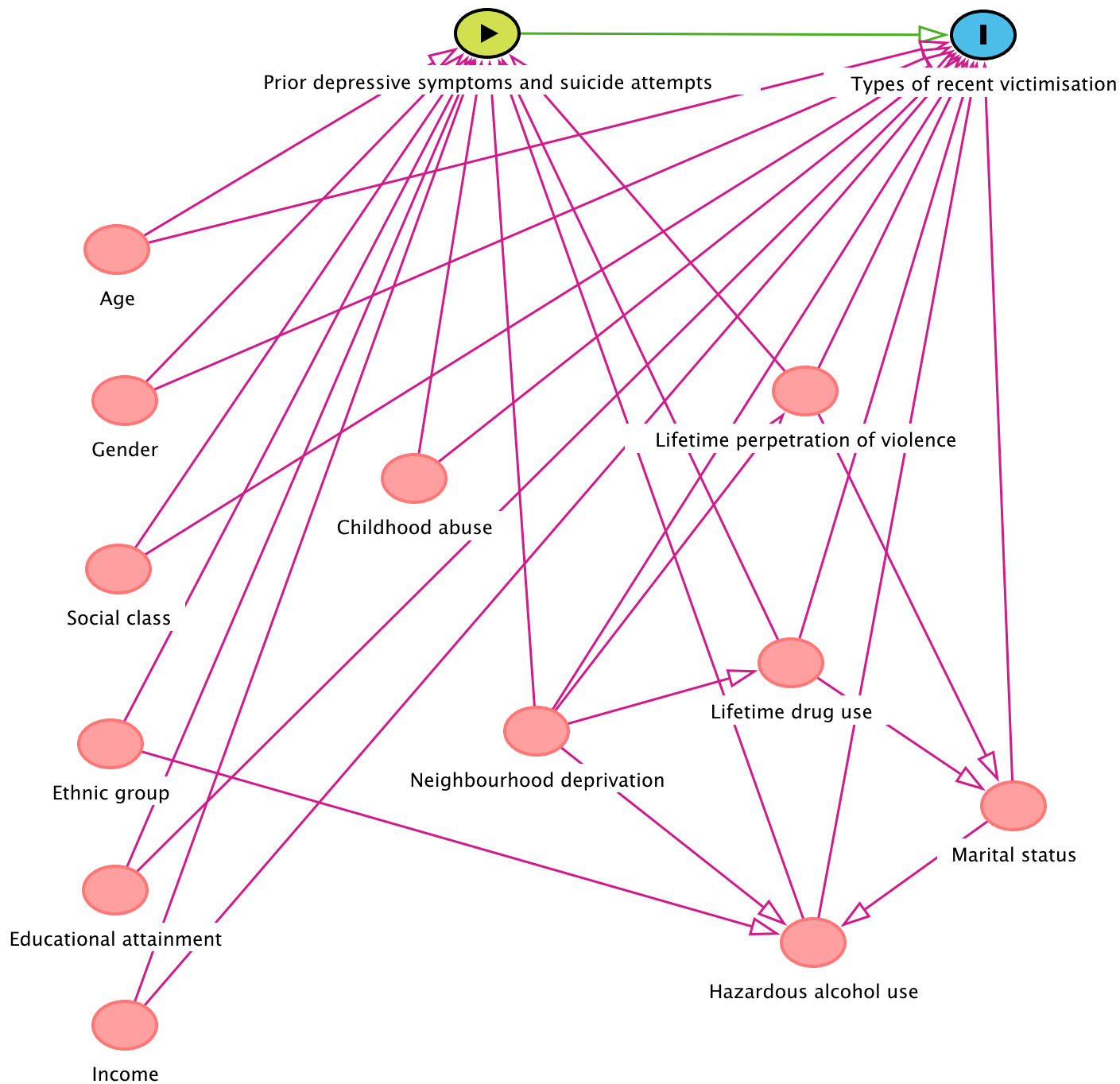

Supplement: Supplementary file 1 [file S0924933820000504sup.zip › S0924933820000504sup002.pdf]
